# Force field-inspired transformer network assisted crystal density prediction for energetic materials

**DOI:** 10.1186/s13321-023-00736-6

**Published:** 2023-07-19

**Authors:** Jun-Xuan Jin, Gao-Peng Ren, Jianjian Hu, Yingzhe Liu, Yunhu Gao, Ke-Jun Wu, Yuchen He

**Affiliations:** 1 Zhejiang Provincial Key Laboratory of Advanced Chemical Engineering Manufacture Technology, College of Chemical and Biological Engineering, Zhejiang University, Hangzhou, 310027 China; 2 Institute of Zhejiang University-Quzhou, Quzhou, 324000 China; 3 Xi’an Modern Chemistry Research Institute, Xi’an, 710065 China; 4 Department of Engineering, University of Cambridge, Cambridge, CB2 1PZ UK; 5 State Key Laboratory of Industrial Control Technology, College of Control Science and Engineering, Zhejiang University, Hangzhou, 310027 China

**Keywords:** Energetic materials, Machine learning, Graph neural networks, Crystal density prediction

## Abstract

Machine learning has great potential in predicting chemical information with greater precision than traditional methods. Graph neural networks (GNNs) have become increasingly popular in recent years, as they can automatically learn the features of the molecule from the graph, significantly reducing the time needed to find and build molecular descriptors. However, the application of machine learning to energetic materials property prediction is still in the initial stage due to insufficient data. In this work, we first curated a dataset of 12,072 compounds containing CHON elements, which are traditionally regarded as main composition elements of energetic materials, from the Cambridge Structural Database, then we implemented a refinement to our force field-inspired neural network (FFiNet), through the adoption of a Transformer encoder, resulting in force field-inspired Transformer network (FFiTrNet). After the improvement, our model outperforms other machine learning-based and GNNs-based models and shows its powerful predictive capabilities especially for high-density materials. Our model also shows its capability in predicting the crystal density of potential energetic materials dataset (i.e. Huang & Massa dataset), which will be helpful in practical high-throughput screening of energetic materials.

**Supplementary Information:**

The online version contains supplementary material available at 10.1186/s13321-023-00736-6.

## Introduction

Machine Learning (ML) is a data-driven method that has gained widespread attention in various fields over the last few decades and shows great potential to predict chemical information with greater precision than traditional methods [1–5]. Supervised ML methods facilitate numerous data to learn the pattern between the molecule and the certain property we need, which is often difficult to give a theoretical or empirical formula. This powerful tool makes it possible to perform high-throughput virtual screening (HTVC), which will significantly accelerate the process of discovering new materials or new drugs [6–8].

Traditionally, the search for novel materials involved conducting a series of time-consuming and labor-intensive experiments and theoretical studies, leading to a very long period of material screening [9]. For example, the development of energetic materials often takes a decade or even more [10]. With the development of computer science, computational simulation was applied to the prediction of molecular properties, such as molecular dynamics (MD) [11] and density functional theory (DFT) [12]. These methods significantly reduce the experimental time and costs of material selection by pre-screening materials based on in silico calculated properties, which could quickly eliminate poor-performing materials without further experimentation. But these molecular simulation methods also have their problems. For example, they are often computationally consuming [13] and require computer clusters; Additionally, these methods need to recalculate all data every time a new environment or target molecule is introduced, even if it is similar to a previous one, meaning that they cannot make use of prior knowledge [9]. To overcome these weaknesses, a variety of methods have been developed. One of the most powerful and popular is the ML method.

Over the past decades, different ML approaches, such as support vector machine (SVM) [14, 15], random forest (RF) [16, 17], and artificial neural network (ANN) [18–20], have been broadly applied in predicting molecular properties and have shown great applicability. These ML methods all use the quantitative structure–property relationships (QSPR) [21] which depends on a large number of molecular descriptors or fingerprints: Coulomb matrices [22], bag of bonds [23], etc., to give a rather accurate prediction about the molecular property, while these molecular descriptors are sometimes hard to obtain. Hence, in order to circumvent the challenge of locating or creating these complex descriptors, it is imperative to identify a simple yet precise representation for the molecules.

In the last few years, a new ML method called graph neural networks (GNNs) [24, 25] has gained more and more attention and become increasingly popular. Since molecules can be represented as graphs (the atoms as nodes and the bonds as edges), by aggregating and updating the features of all the atoms and bonds, GNNs can automatically learn the features of the molecule from the graph, which significantly reduces the time we find and build the molecular descriptors. However, despite the convenience and promising expectations of the ML, the application to energetic materials property prediction is still at the initial stage, due to an insufficient amount of data [26, 27].

Energetic materials represent a class of materials capable of releasing large amounts of chemical energy stored inside the molecular structure. Typical energetic materials include explosives, propellants, fuels, pyrotechnic compositions, etc. which are widely used not only in military applications but also in civil engineering and space exploration (e.g., mining and rocket propellant) [28]. Crystal density is an important property of energetic materials, which is highly related to other detonation performance characteristics. e.g., the detonation pressure is approximately proportional to the square of the density [29]. One of the main criteria for the evaluation of promising energetic materials is ‘high’ density, which typically refers to a density greater than 1.8 g/cm^3^ [30].

Traditionally, group additivity [31, 32] and some empirical methods [33, 34] were performed to predict the crystal density of energetic materials. While in recent years, ML-based methods have emerged as a promising approach for predicting crystal density with enhanced accuracy and reliability. Fathollahi et al. [35] conducted a study on 26 energetic cocrystals, in which they extracted three molecular descriptors from the optimized chemical structures. They predicted the densities of these cocrystals using an ANN with a test precision up to 0.9918. Despite the small amount of data, this still shows the great potential of ML-assisted methods in the crystal density prediction of energetic material. Casey et al. [36] raised a 3D convolution neural network (CNN) using charge density and electrostatic potential as the represented feature, which got a high accuracy prediction in the dataset screening the possible energetic materials from the GDB database [37–39]. Yang et al. [40] noticed the difficulty and cumbersomeness of extracting these molecular descriptors and started to use GNNs to learn these descriptors merely from its topology. They found that GNNs-based model could achieve higher accuracy and lower computational resource with respect to other traditional ML methods. Recently, more and more ML methods have been used in the prediction of crystal density. Then, Nguyen et al. [41] use an improved GNNs model called Directed Message Passing Neural Networks (D-MPNNs), which is raised by Yang et al. [42], which utilizes the directed graph, rather than the traditional undirected graph, to represent the molecule and update nodes and edges feature using message passing algorithm. This model outperforms other ordinary models, SVM, RF, and Partial Least Squares Regression (PLSR), achieving a more accurate result.

**Fig. 1:**
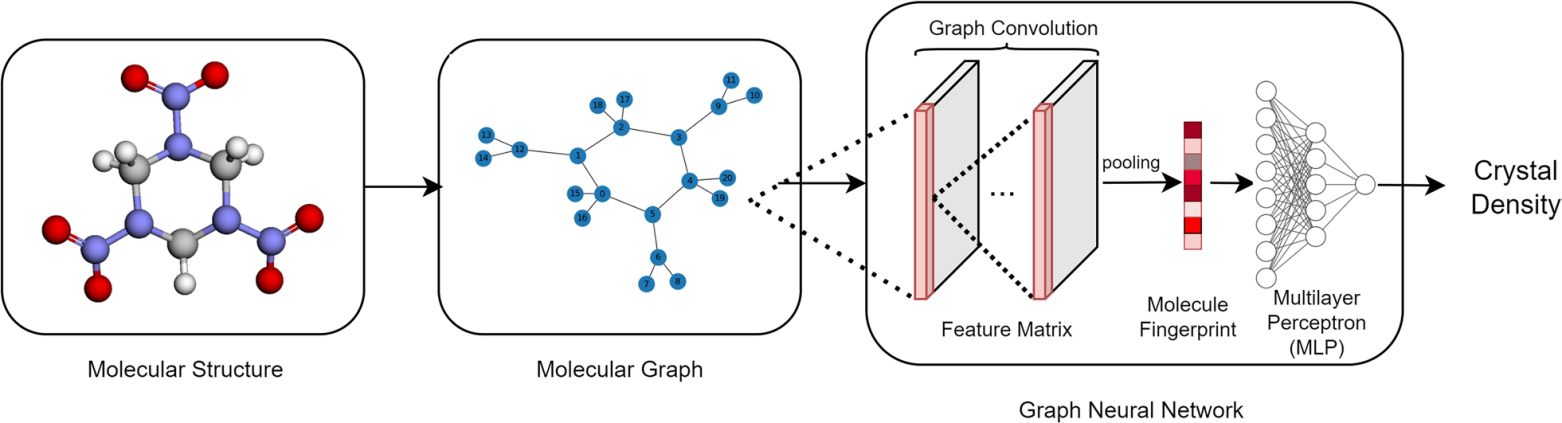
Flowchart for predictive screening process of energetic material density using GNNs

From the example above, GNNs uses none of a priori knowledge, only the topology structure of each molecule, but can achieve a higher predicting accuracy. Thus, it could be the most promising method for predicting the crystal density of energetic materials. A typical process of density prediction using GNNs is shown in Fig. 1. However, most available GNNs model utilizes only 2D molecular descriptors to present the molecule, leading to large biases in describing the 3D caged molecules like CL-20 family molecules (e.g. Hexanitrohexaazaisowurtzitane) or cubane family molecules (e.g. Octanitrocubane).

Therefore, in this work, the main aim is to further improve the prediction accuracy of the density of 3D molecules, and minimize errors, particularly in the high-density region of interest, i.e. the density higher than 1.8 g/cm^3^. We adopt force field-inspired neural network (FFiNet) [43], a 3D-aware GNNs developed by our group using force fields to calculate the energy of bonds, angles, dihedral, and non-bonded interaction as attention scores, which is able to give more accurate molecular information when dealing with 3D molecules having the spatial caged structures. Moreover, the self-attention mechanism from Transformer is used to replace the axial attention in original model, to give a global representation of the force fields terms, showing a great accuracy improve in the crystal density prediction task. This modified model is called force field-inspired Transformer networks (FFiTrNet), and the detailed information of FFiTrNet will be illustrated later.

## Methodologies

### Data set

One of the biggest problems between ML and energetic materials is the lack of sufficiently large datasets. In order to get a prediction with high accuracy, a large, diverse, and accurate dataset is needed so that the model learns the pattern in the data set properly. In other prediction tasks, the common size of other public datasets is over 1000, for example, BBBP (*N* = 2039), Lipophilicity (*N* = 4200), QM7 (*N* = 7160) in MoleculeNet [44]. This is much bigger than the current accessible energetic material datasets, making it hard to give a reasonable prediction and may lead to overfitting in the given datasets [26, 27]. In order to generalize our prediction model, we need to find a larger dataset containing molecules with the same molecular frameworks and functional groups as the energetic materials. In this work, we curated the dataset from the Cambridge Structural Database (CSD) [45], which contains more than 1.1 M organic and metal–organic crystal structure data, allowing big-data searching and screening. Moreover, each structure within the CSD undergoes extensive validation and cross-checking via automated workflows and through manual curation by expert chemists and crystallographers. This guarantees data accuracy, consistency, and high quality within the CSD, making the ML model more trustful.

The candidates are curated through the following criteria: (1) those only consist of carbon (C), hydrogen (H), oxygen (O), and nitrogen (N) atoms; (2) those have at least one of any carbon–nitrogen bonds; (3) those don’t belong to ionic or co-crystal compounds; (4) those are able to make 3D conformation in further operation. By initially screening the data using the steps above, we established a dataset with 12,072 compounds containing CHON elements with their Simplified Molecular-Input Line-Entry System (SMILES) strings and crystal density. This will satisfy our need for a large enough dataset and can be used to train our model efficiently.

### Data preprocessing and featurization

First, we grab the 3D conformation of the molecules that contain atoms’ position information using RDkit toolkit [46] from SMILES strings of data. RDkit toolkit is a widely used open-source machine-learning software providing a collection of cheminformatics for descriptor and fingerprint generation, 2D and 3D molecular operations, etc. The fast ETKDG method [47] from RDkit is applied to generate atom positions. After obtaining the 3D molecular graph and its positional information, we extract atom features foreach atom in molecules, and the atom feature was listed in Table 1.

**Table 1:** The atom features used in the FFiTrNet^a^

Feature	Description	Size
Atom type	The type of the atom	38
Atom degree	The number of directly-bonded neighbors	6
Chiral type	The chiral type of the atom: unspecified, tetrahedral CW, tetrahedral CCW, or other	4
Hs number	The total number of hydrogens attached to the atom	6
Hybridization	The hybridization type of the atom: unspecified, *s*, *sp*, *sp*2*d*, *sp*3, *sp*3*d*, *sp*3*d*2, or other	8
Aromatic	Whether an atom belongs to the aromatic ring	1
Atomic mass	The mass of the atom	1
Hydrogen bond	Whether an atom accepts electrons or donates electrons	2

^a^All features are one-hot encodings except for atomic mass

### Model framework

In this work, we adapt FFiNet as the main framework of the model to learn the feature from molecular topology. The position information in the 3D conformer of each molecule is fed into the model. This information is then used to calculate the distance, angle, and dihedral information from all the neighbors or 2-hop, and 3-hop neighbors of each atom. According to the traditional force field theory [48], the potential energy could be written as:

1 $${E}_{total}={E}_{bond}+{E}_{angle}+{E}_{tor}+{E}_{non-bonded}$$

By expanding the bond term of energy in empirical model:

2 $${E}_{\mathrm{bond}}=\sum_{\mathrm{bonds}}{K}_{r}{\left(l-{l}_{\mathrm{eq}}\right)}^{2} ={f}_{bond}\left(l,{l}^{2}\right)$$

3 $${E}_{\mathrm{angle}}=\sum_{\mathrm{angles}}{K}_{\theta }{\left(\theta -{\theta }_{\mathrm{eq}}\right)}^{2}={f}_{angle}\left(\theta ,{\theta }^{2}\right)$$

4 $${E}_{tor}=\sum_{\mathrm{dihedrals}}\frac{{V}_{\phi ,1}}{2}\left[1+\mathrm{cos}\left(\phi +{f}_{\phi ,1}\right)\right]+\frac{{V}_{\phi ,2}}{2}\left[1-\mathrm{cos}\left(2\phi +{f}_{\phi ,2}\right)\right]+\frac{{V}_{\phi ,3}}{2}\left[1+\mathrm{cos}\left(3\phi +{f}_{\phi ,3}\right)\right]={f}_{tor}\left(cos\phi ,cos2\phi ,cos3\phi ,sin\phi ,sin2\phi ,sin3\phi \right)$$

5 $${E}_{non-bonded}=\sum_{i}\sum_{j}\left[\frac{{q}_{i}{q}_{j}}{{r}_{ij}}+4{\epsilon }_{ij}\left(\frac{{\sigma }_{ij}^{12}}{{r}_{ij}^{12}}-\frac{{\sigma }_{ij}^{6}}{{r}_{ij}^{6}}\right)\right]{f}_{ij}={f}_{non-bonded}\left({r}^{-1},{r}^{-6},{r}^{-12}\right)$$

where ${K}_{r}$, ${K}_{\theta }$, ${V}_{\phi ,n}(n=\mathrm{1,2},3)$, ${f}_{ij}$, ${\epsilon }_{ij}$ are all force constants; ${f}_{\phi ,n}(n=\mathrm{1,2},3)$ are dihedral phase; $l$, $\theta$, $\phi$ and $r$ represent the bond length, angle, dihedral angle, and the distance between non-bonded atoms respectively; ${l}_{eq}$, ${\theta }_{eq}$, and ${\sigma }_{ij}$ are the value of bond, angle, or non-bonded atomic distance when the corresponding energy term is considered zero as the reference value; ${q}_{i}$ and ${q}_{j}$ are the atomic charges and $f(\cdot)$ is a general linear function. The detailed information can be found in the Additional file 1 and our previous work [43].

In this model, we only calculate the non-bonded term for the 2-hop and 3-hop neighbors of each atom and add it to the corresponding angle and torsion terms as a part of energy. Then a one-layer linear transformation is used as the linear function $f(\cdot)$ also as an embedding layer to facilitate the next operation. These energy embedding terms are treated separately after attention operation and then combined through axial attention to give an output embedding.

Because the axial attention treats its inputs separately, and simply sums up the output to further operation, there’s no information interaction between different energy terms. This doesn’t match the reality for each energy term is highly affected by each other. So, we further improve the performance by introducing the encoder layer from Transformer to replace the axial attention, to help us mix these energy terms’ information together.

**Fig. 2:**
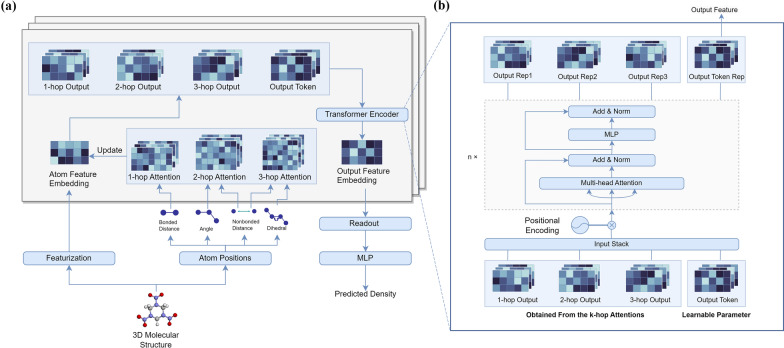
**a** Structure diagram of the FFiTrNet model. Transformer encoder is used to replace the axial attention in the origin FFiNet. **b** The detailed structure of the Transformer encoder in FFiTrNet. A special output token is introduced as one of its inputs to aggregate all the information of three k-hop outputs

Transformer was first introduced in 2017 by Vaswani et al. [49] to solve natural language processing but quickly show great potential in a wide field of ML. Its encoder layer adopts the self-attention mechanism that allows the model to attend to different parts of the input sequence while processing each position. After using the k-hop (k = 1, 2, 3) attention to update the embedding feature, we got 1-hop, 2-hop, and 3-hop outputs, stacked with a special output token as the learnable parameter, which is inspired by the same concept in vision Transformers (ViTs) [50]. Then they are fed into the Transformer encoding layer to get the same amount of output representations of the same length, and one of the representations corresponding to the special output token is picked as the final output to go through further operation. The detailed structure of the FFiTrNet is shown in Fig. 2. In there, positional encoder is considered optional because there’s no obvious positional relation in these four outputs, and one layer of Transformer encoding layer is good enough for this case.

### Model evaluation

We adopt three different metrics to evaluate the regression model, like mean absolute error (MAE), root mean square error (RMSE), and coefficient of determination $({R}^{2})$:

6 $$\mathrm{MAE}=\frac{1}{\mathrm{N}}\sum_{i=1}^{N}|{\rho }_{i}^{true}-{\rho }_{i}^{pred}|$$

7 $$\mathrm{RMSE}=\sqrt{\frac{1}{\mathrm{N}}\sum_{i=1}^{N}{\left({\rho }_{i}^{true}-{\rho }_{i}^{pred}\right)}^{2}}$$

8 $${R}^{2}=1-\frac{\sum_{i=1}^{N}{\left({\rho }_{i}^{true}-{\rho }_{i}^{pred}\right)}^{2}}{\sum_{i=1}^{N}{\left({\rho }_{i}^{true}-\overline{\rho }\right)}^{2}}$$

where ${\rho }^{true}$ is the true density value of the sample, ${\rho }^{pred}$ is the predicted density value of the sample, $\overline{\rho }$ is the average density value of the true density.

## Results and discussion

There is a widely accepted standard for evaluating predictions of crystal density: a prediction with an absolute error less than 0.03 g/cm^3^ is considered an “excellent” prediction; the absolute error between 0.03 and 0.05 g/cm^3^ is considered “informative”; the absolute error between 0.05 and 0.10 g/cm^3^ is considered “barely useful”; and the absolute error greater than 0.10 g/cm^3^ is considered “deceptive” [51, 52]. In this work, as our improved FFiTrNet model is based on the GNNs which only uses the molecules’ topology, we mainly compare it to other GNNs like graph attention networks (GATv2) and D-MPNNs, which have been proven to be highly accurate in predicting the crystal density of energetic materials by Nguyen et al. [41]. Also, we adapt RF which uses molecular descriptors of QSPR as the input to show that the GNNs-learnt descriptor could outperform the man-made molecule descriptors. Moreover, in order to validate the effects of Transformer encoder, the result of the original FFiNet is also listed.

### Overall testing evaluation

We first use the dataset (N = 12,072) that was curated from CSD database, randomly splitting the data into training, validation, and test dataset with a ratio of 0.8:0.1:0.1. We performed three independent runs with different random seeds for each model. The result is shown in Table 2, presented in the form of “mean ± standard deviation” of the three runs after the hyperparameter optimization. Moreover, all the models are using the same training strategy for a fair comparison.

**Table 2:** The test MAE, RMSE and *R*^2^ for each model

Models	MAE (g/cm^3^)	RMSE (g/cm^3^)	*R*^2^
RF	0.0367 ± 0.0010	0.0514 ± 0.0019	0.8886 ± 0.0094
GATv2	0.0330 ± 0.0005	0.0466 ± 0.0041	0.9101 ± 0.0162
D-MPNNs	**0.0313 ± 0.0008**	*0.0463 ± 0.0049*	*0.9113 ± 0.0146*
FFiNet	0.0330 ± 0.0013	0.0479 ± 0.0037	0.9005 ± 0.0127
FFiTrNet	**0.0313 ± 0.0004**	**0.0448 ± 0.0022**	**0.9170 ± 0.0141**

The best results are marked in bold, and the second-best results are italicized

**Fig. 3:**
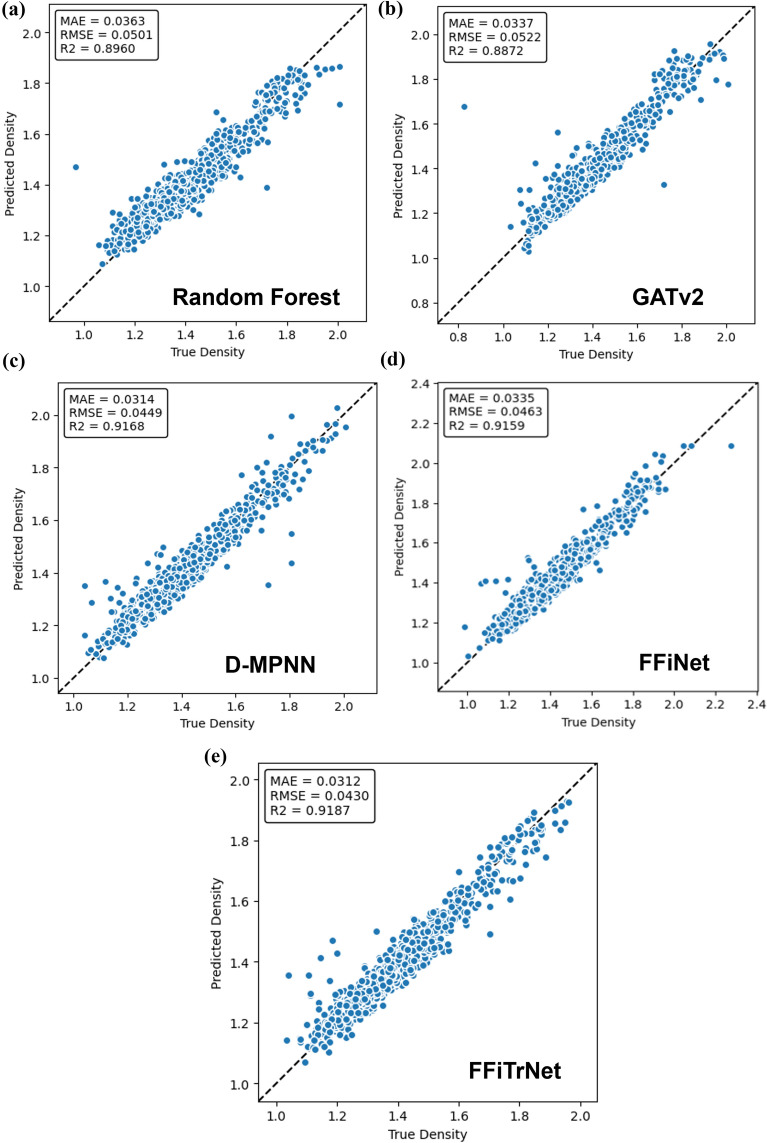
The parity plot of true density versus predicted density of each model. All the data in the graph is from the test dataset. **a** Random Forest; **b** GATv2 **c** D-MPNNs; **d** FFiNet; **e** FFiTrNet

Based on the results from Table 2 and Fig. 3, we can see that RF which employs descriptors created by humans, exhibits the poorest performance in this prediction task. The number of feature used in RF is 208, which is rather easy to obtain, so this finding demonstrates that GNNs have already developed descriptive features that possess more expressive power than these easily obtainable RDkit molecular descriptors. For the GNNs model, our original FFiNet performs worse than the GATv2 and D-MPNNs when dealing with crystal density. But after introducing the Transformer encoder into the FFiNet model, FFiTrNet’s performance has been improved considerably, reaching a slightly better result than the D-MPNNs. This shows that Transformer encoder does make the k-hop information more expressive and the prediction more accurate.

However, because of the distribution of our CSD curated dataset, in which only 322 out of 12,072 data points’ density are within our interested region, i.e. over 1.8 g/cm^3^, most of the prediction errors do not affect the final decision of screening. This is to say that if the true crystal density of one molecule is pretty much lower than 1.8 g/cm^3^, the higher prediction accuracy is not much important because it will be quickly screened out of the promising candidates’ list. Thus the prediction accuracy in different density regions should be treated differently, especially those within and near our interested density region.

**Fig. 4:**
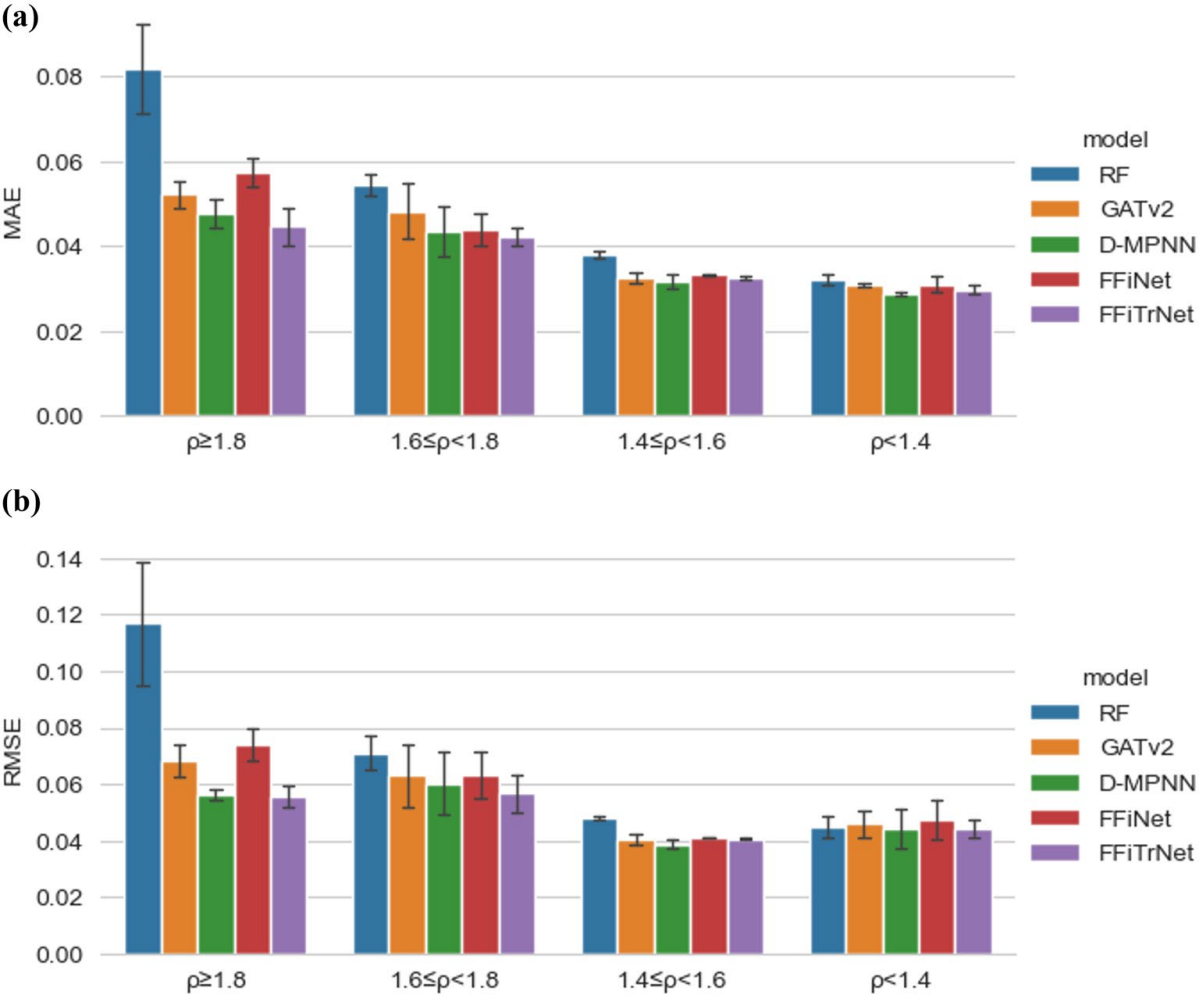
Comparison of each model’s performance in different density regions. **a** MAE; **b** RMSE. Our model outperforms other models in high-density regions and is second only to the D-MPNNs in low-density regions in which high accuracy is not important. The unit of crystal density in graph is g/cm^3^

In order to evaluate the performance of our model, we split the test dataset of each model above into 4 regions: (1) density higher than 1.8 g/cm^3^, our interested high-density region. A bunch of modern energetic materials, 1,3,5-Trinitro-1,3,5-triazinane (RDX, 1.806 g/cm^3^), 1,3,5,7-Tetranitro-1,3,5,7-tetrazocane (HMX, 1.91 g/cm^3^), hexanitrohexaazaisowurtzitane (CL-20, 2.044 g/cm^3^), etc. fall in this region, therefore accurate prediction is highly desirable; (2) density between 1.6 and 1.8 g/cm^3^, near the interested region. Conventional energetic materials such as 2,4,6-trinitrotoluene (TNT, 1.654 g/cm^3^) fall in it, and should have a certain level of accuracy; (3) density between 1.4 g/cm^3^ and 1.6 g/cm^3^, not that much important; (4) density lower than 1.4 g/cm^3^, out of consideration. Then, we list out the test MAE, RMSE, and *R*^2^ of each region, as shown in Table 3 and Fig. 4. Because of the small amount of data in regions 1 and 2, *R*^2^ is pretty small in these regions, making it meaningless and not comparable between each model.

**Table 3:** The test MAE and RMSE for each model in different density region

Density region	Models	MAE (g/cm^3^)	RMSE (g/cm^3^)
1) ρ ≥ 1.8 g/cm^3^	RF	0.0818 ± 0.0107	0.1169 ± 0.0219
	GATv2	0.0522 ± 0.0032	0.0681 ± 0.0057
	D-MPNNs	*0.0476 ± 0.0033*	*0.0564 ± 0.0020*
	FFiNet	0.0573 ± 0.0034	0.0739 ± 0.0056
	FFiTrNet	**0.0446 ± 0.0045**	**0.0556 ± 0.0036**
2) 1.6 g/cm^3^ ≤ ρ < 1.8 g/cm^3^	RF	0.0544 ± 0.0024	0.0710 ± 0.0060
	GATv2	0.0482 ± 0.0064	0.0631 ± 0.0112
	D-MPNNs	*0.0436 ± 0.0059*	*0.0604 ± 0.0113*
	FFiNet	0.0439 ± 0.0037	0.0631 ± 0.0081
	FFiTrNet	**0.0422 ± 0.0021**	**0.0567 ± 0.0068**
3) 1.4 g/cm^3^ ≤ ρ < 1.6 g/cm^3^	RF	0.0380 ± 0.0008	0.0479 ± 0.0006
	GATv2	0.0326 ± 0.0012	*0.0404 ± 0.0018*
	D-MPNNs	**0.0316 ± 0.0015**	**0.0387 ± 0.0016**
	FFiNet	0.0331 ± 0.0003	0.0412 ± 0.0002
	FFiTrNet	*0.0325 ± 0.0003*	0.0407 ± 0.0004
4) ρ < 1.4 g/cm^3^	RF	0.0321 ± 0.0014	0.0449 ± 0.0040
	GATv2	0.0308 ± 0.0003	0.0459 ± 0.0047
	D-MPNNs	**0.0287 ± 0.0006**	*0.0443 ± 0.0067*
	FFiNet	0.0309 ± 0.0018	0.0473 ± 0.0069
	FFiTrNet	*0.0297 ± 0.0011*	**0.0443 ± 0.0029**

The best results are marked in bold, and the second-best results are italicized

**Fig. 5:**
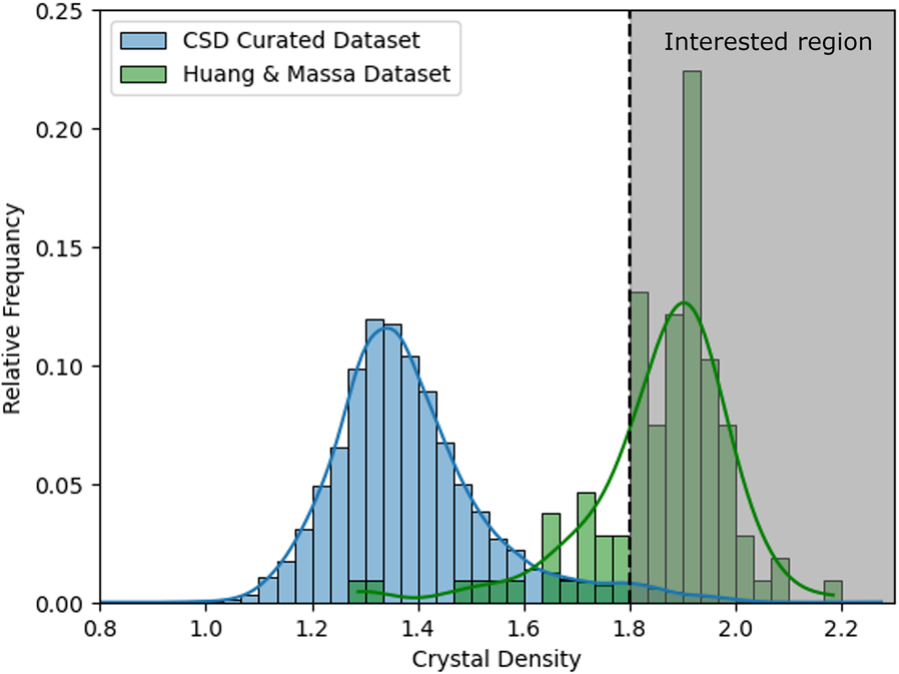
The relative distribution of two datasets. Crystal density higher than 1.8 g/cm^3^ is our interested region. The ratio of the interested data is 0.0263 (318 out of 12,072) for CSD curated dataset and 0.798 (87 out of 109) for Huang & Mass dataset, showing a great bias of these two datasets

From the results above, FFiTrNet outperforms the other models in regions 1 and 2, having the lowest MAE, RMSE and highest* R*^2^. For regions 3 and 4, which make up most of the CSD curated dataset, as shown in Fig. 5, FFiTrNet performs worse than the D-MPNNs but is still in second place. This suggests our model could give a better prediction dealing with the high-density materials, and D-MPNNs having quite the same overall accuracy as FFiTrNet though, has less ability to handle the data point out of main dataset part, indicating overfitting in the main part of the dataset.

### Crystal density predicting task for potential energetic materials dataset

As said before, the main problem with using ML in predicting the energetic materials’ crystal density is the lack of large datasets. But after enlarger the dataset to the CHNO molecules, it is possible to use the curated-dataset-trained model to predict a small energetic material dataset. In there, we use another small dataset from Huang & Massa [53], who obtain explosive properties against 109 putative energetic materials and thereby produce their energetic characteristics, including the crystalline density, using quantum chemical calculations. In Huang & Massa dataset, most of the data (87 out of 109, with a ratio of 0.798) have a density greater than 1.8 g/cm^3^, the ratio of the interested data is much higher than that of the CSD curated dataset (318 out of 12,072, with a ratio of 0.0263). The data distribution of the two datasets is shown in Fig. 5. Due to the high-density distribution of Huang & Massa dataset, the prediction error for this dataset will be more practical, without error distortion from the low-density data. So the prediction accuracy for Huang & Massa dataset should be considered more important for the real screening process. Before using Huang & Massa dataset as the test data, we first removed the training data points that appear in both two datasets, to make sure all the test data are unseen, so the test result will be comparable and reliable.

All the results, in the form of mean ± standard deviation of three independent runs, are shown in Table 4.

**Table 4:** The test MAE, RMSE and* R*^2^ for each model using Huang & Massa dataset as test dataset

Models	MAE (g/cm^3^)	RMSE (g/cm^3^)	*R*^2^
RF	0.0620 ± 0.0004	0.0964 ± 0.0006	0.5144 ± 0.0058
GATv2	*0.0515 ± 0.0024*	*0.0631 ± 0.0022*	*0.7915 ± 0.0145*
D-MPNNs	0.0602 ± 0.0013	0.0794 ± 0.0010	0.6704 ± 0.0085
FFiNet	0.0561 ± 0.0020	0.0712 ± 0.0023	0.7346 ± 0.0172
FFiTrNet	**0.0489 ± 0.0012**	**0.0604 ± 0.0012**	**0.8092 ± 0.0077**

The best results are marked in bold, and the second-best results are italicized

**Fig. 6:**
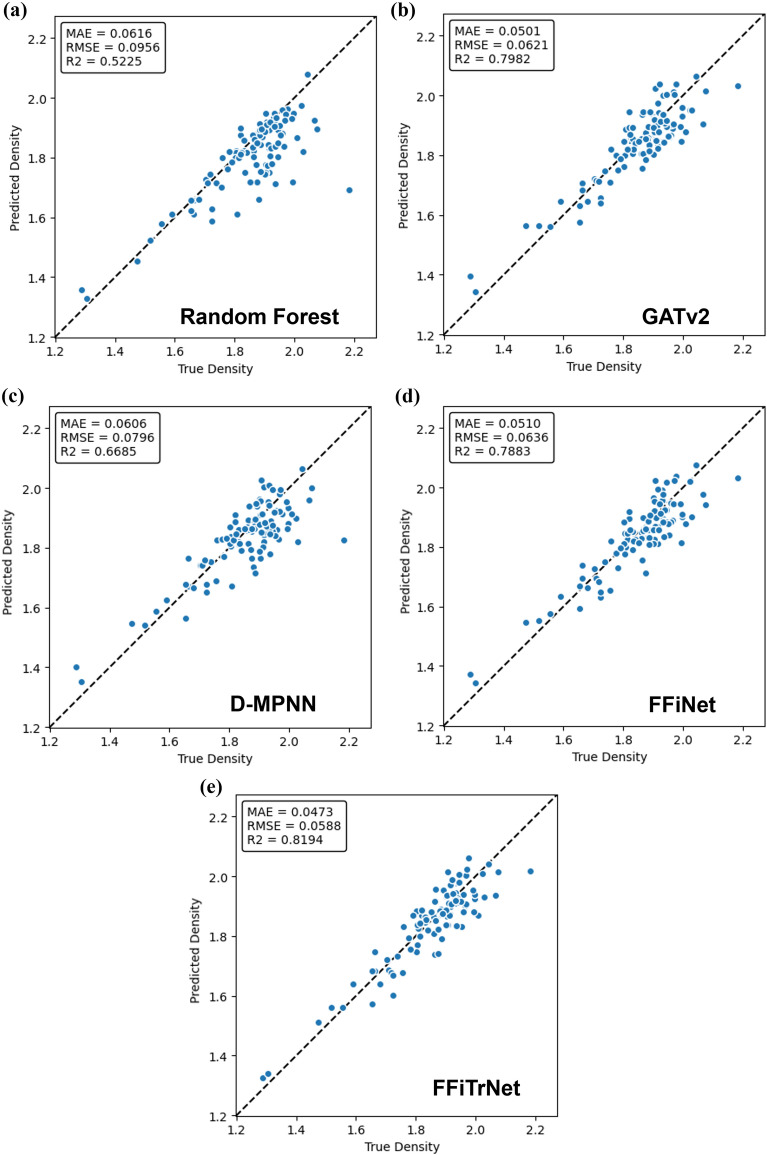
The parity plot of true density versus predicted density of each model using Huang & Massa dataset as test dataset **a** Random Forest; **b** GATv2; **c** D-MPNN **d** FFiNet; **e** FFiTrNet

It can be seen from Table 4 and Fig. 6, our FFiTrNet model outperforms other models. Although there’s an accuracy drop compared to Table 3, where the MAE is 0.0489 g/cm^3^ compared to 0.0446 g/cm^3^ for the region that the density is higher than 1.8 g/cm^3^, FFiTrNet still has a relatively good prediction, which is considered “informative” as it’s lower than 0.05 g/cm^3^. This accuracy drop may be caused by the different molecule types in Huang & Massa dataset. Different from CSD curated dataset that only contains the CHNO compound, 23 molecules in Huang & Massa dataset contain fluorine atoms, which is completely unseen in the training process, thus the effect of fluorine atoms on the crystal density is not learned, making the FFiTrNet less accurate. Interestingly, D-MPNNs’ performance on the Huang & Massa dataset is relatively poor, though its overall test error for CSD curated dataset is close to FFiTrNet. It might be that D-MPNNs overfit in the main part of CSD curated dataset, whose density is mostly below 1.6 g/cm^3^, as shown in the discussion of Table 3. For the model with simpler structures, like GATv2 and RF, the overfitting is not significant, so they don’t suffer from this accuracy drop and even perform better.

### Model interpolation through the molecular structure

In Huang & Massa dataset, all 109 energetic materials are divided into 10 distinct compound families (or groups). The families are labeled according to some characteristic chemical like CL-20 and HMX, or structural feature uniting members of the families. By listing out the MAE of each group, we can further investigate the relationship between molecular structure and model accuracy.

**Fig. 7:**
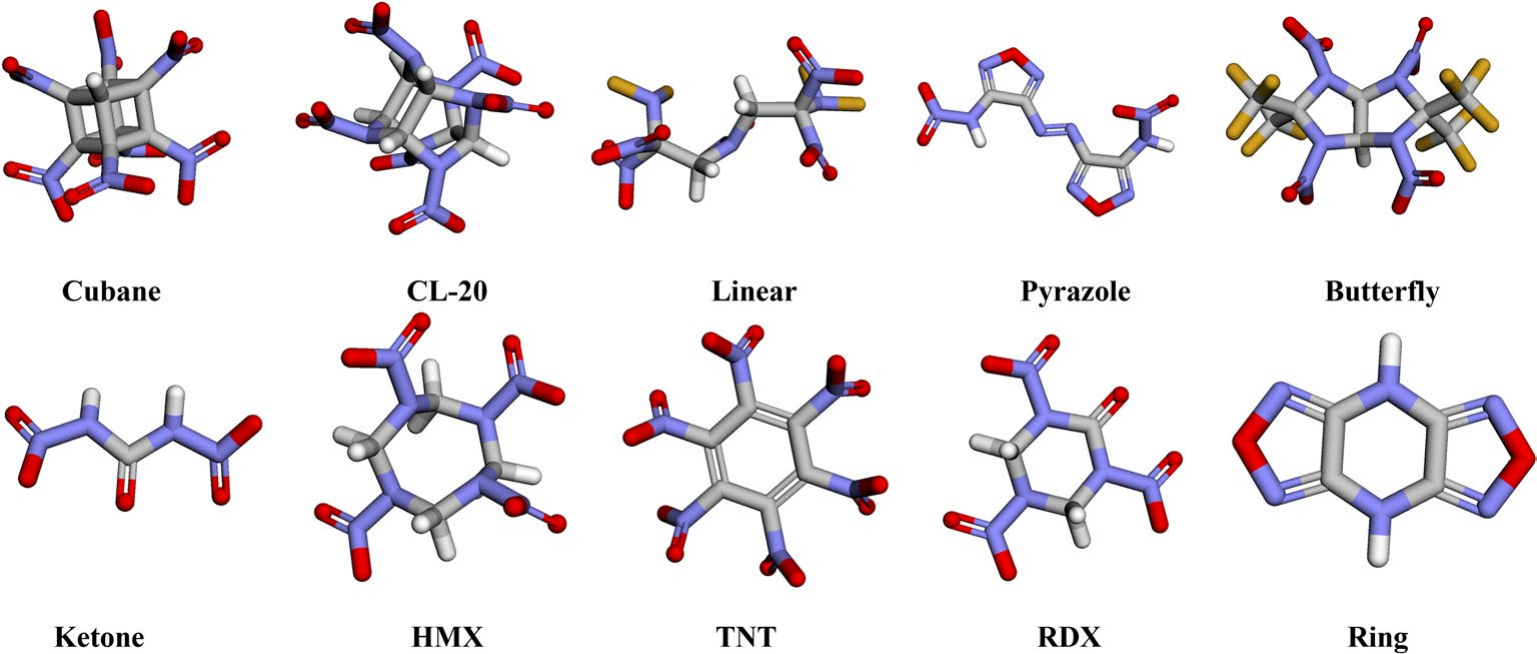
The instance of each family in Huang & Massa dataset, shown in 3D form. The carbon atom (C) is shown in grey color; the oxygen atom (O) is shown in red color; the nitrogen atom (N) is shown in blue color and the fluorine atom (F) is shown in yellow color

In Table 5, all the mean absolute errors of each molecule family are listed, and Fig. 7 gives the 3D molecular structure of one example from each family to help better understand the structural difference between each family.

**Table 5:** The Mean Absolute Errors (g/cm^3^) of Each Molecule Family Using Huang & Massa Dataset as Testing

Molecule families	RF	GATv2	D-MPNN	FFiNet	FFiTrNet
Cubane	0.1013	*0.0548*	0.0662	0.0620	**0.0449**
CL-20	0.0694	0.0616	0.0673	0.0850	**0.0601**
Linear	0.0994	*0.0494*	0.0684	0.0513	**0.0480**
Pyrazole	0.0573	0.0505	0.0481	**0.0466**	*0.0470*
Butterfly	0.1146	0.0736	0.0874	0.0699	**0.0693**
Ketone	0.1108	0.0521	0.0517	0.0653	**0.0464**
HMX	0.0871	0.0420	0.0671	0.0475	**0.0399**
TNT	0.0666	*0.0422*	0.0498	**0.0410**	0.0475
RDX	0.0930	**0.0502**	0.0641	0.0644	*0.0572*
Ring	0.0744	0.0620	**0.0475**	0.0680	*0.0593*

The best results are marked in bold, and the second-best results are italicized

All data in the table are the average of three independent runs

From Table 5, comparing FFiNet and FFiTrNet models, we can see that after adding Transformer encoder, FFiTrNet model shows performance improvement in most of the families. When compared to other models, FFiTrNet also has the lowest prediction error in most of the molecular families, especially those molecules with complex 3D structures, such as cubane and CL-20 families. Also, because the cage structure usually has a higher crystal density and energy density, having attracted much attention in the energetic materials field [54], the prediction accuracy for caged structures should be high on the list of priorities. This could also explain why our model performs better in the high-density region, because they have a higher ratio of these caged molecules. And the higher accuracy in the unseen data shows our model gets less overfit in the training dataset, learning the more fundamental pattern inside the molecule graph. The promising result in the high-density and out-of-distribution dataset makes our model is powerful tool to predicting and screening for the potential energetic materials.

## Conclusions

Crystal density is an important property of energetic materials, but applying ML methods to predict energetic materials’ crystal density still face the problem of insufficient data. In this work, we curate a relatively big dataset from CSD containing 12,072 data of CHON compounds with merely SMILES string and crystal density to overcome this problem. New 3D-aware GNNs models FFiNet and its upgraded version FFiTrNet are then trained and tested in this CSD curated dataset. Our FFiTrNet model outperforms other ML models, RF, GATv2 and D-MPNN, especially in the high-density region, which has more importance in the practical screening process, showing FFiTrNet overfits less in the low-density region and has more generalizability. After training the models on the CSD curated dataset, we use this pretrained model to predict the potential energetic materials dataset: Huang & Massa dataset, showing great performance in this out-of-distribution dataset. Finally, we further investigate the effect of some certain molecular structure on the models, FFiTrNet using 3D conformation of molecules could give a more accurate prediction for cage structure, which is the promising searching area of the energetic materials. But also, the deeper interpolation of the model is needed for a better understanding of how force field terms work in predicting the crystal density of different molecule structures, which would be a tough and tricky task, due to the complexity of our model.

All of these results prove that FFiTrNet will be an effective model in predicting the crystal density and screening for new energetic materials. Our model could also be applied to other properties of the energetic materials, such as explosive energy and impact sensitivity, which will be used in the further screening process. Moreover, because our model uses only the SMILES strings of the molecules as the inputs and no a priori knowledge is needed to predict the crystal density, it can be easy to incorporate new molecules into this model, even if we have limited knowledge about them. This helps us to apply this model more simply to the next screening stage, like molecular generation [55, 56] of energetic materials, in which most of the generated molecules will be completely new and unknown. Therefore, our FFiTrNet sets a strong foundation for accelerating the screening of effective energetic materials and for in silico design of new energetic materials, utilizing the molecular generation technique.

## Supplementary Information

**Additional file 1.** Additional model detail, Rdkit feature, and model comparison for small dataset.

## Data Availability

All of the methods are implemented in Python. Source code and dataset is available at GitHub page: https://github.com/jjx-2000/FFiTrNet.
