# Supplementary material for: Force field-inspired transformer network assisted crystal density prediction for energetic materials
Source: J Cheminform. 2023 Jul 19;15:65. doi: 10.1186/s13321-023-00736-6 (PMC10355066; doi:10.1186/s13321-023-00736-6)
Supplement: Supplementary file 1 — Additional file 1. Additional model detail, Rdkit feature, and model comparison for small dataset. [file 13321_2023_736_MOESM1_ESM.docx]

**Supplementary Information for:**

**Force Field-Inspired Transformer Network Assisted Crystal Density Prediction for Energetic Materials**

Jun-Xuan Jin^1,2^, Gao-Peng Ren^1,2^, Jianjian Hu^3^, Yingzhe Liu^3^, Yunhu Gao^4^, Ke-Jun Wu*^1,2^, Yuchen He*^5^

^1^Zhejiang Provincial Key Laboratory of Advanced Chemical Engineering Manufacture Technology, College of Chemical and Biological Engineering, Zhejiang University, Hangzhou 310027, China

^2^Institute of Zhejiang University-Quzhou, Quzhou 324000, China

^3^Xi’an Modern Chemistry Research Institute, Xi’an 710065, China

^4^Department of Engineering, University of Cambridge, Cambridge, CB2 1PZ, UK

^5^State Key Laboratory of Industrial Control Technology, College of Control Science and Engineering, Zhejiang University, Hangzhou, 310027, China

*Corresponding author: KeJun Wu, [K.Wu@zju.edu.cn](mailto:K.Wu@zju.edu.cn); Yuchen He, [hyc19940615@zju.edu.cn](mailto:hyc19940615@zju.edu.cn)

# Model details

## FFiTrNet

For the detailed structure of FFiNet is illustrated in our previous work^1^, we only introduce the detailed information of Transformer encoder in FFiTrNet.

We adopt the TransformerEncoder in PyTorch using the concept of output token from the class token in vision Transformers (ViTs)^2^. The output token is a learnable parameter served as the output representation after the Transformer encoder, helping to integrate the information of k-hop outputs (k = 1, 2, 3). The hyperparameter of the searching space for the Transformer encoder is listed in Table 1.

Table 1 Hyperparameters optimized for Transformer encoder in FFiTrNet

| Hyperparameters | Search space |
| --- | --- |
| Feed forward dimension | 128, 256, 512 |
| Number of encoder layers | 1, 3, 6 |
| Number of heads | 2, 4, 8 |
| Dropout | 0.1, 0.3, 0.5 |
| Positional encoder | On, Off |

## Other models

Scikit-learn^3^ is used for the construction of random forest and hyperparameter tuning. The hyperparameter searching space is listed in Table 2.

Table 2 Hyperparameters optimized for random forest

| Hyperparameters | Search space |
| --- | --- |
| Number of estimators | 200, 400, 600, 800, 1000, 1200, 1400, 1600, 1800,2000 |
| Max features | 10, 20, 30, 40, 50, 60, 70, 80, 90, 100, 110, None |
| Max depth | 2, 4, 8 |
| Min samples split | 2, 5, 10 |
| Min samples leaf | 1, 2, 4 |
| bootstrap | On, Off |

For the GATv2, we use the same model structure and same search space as in our previous work. For the D-MPNN, we removed the feature scaling and scheduler, setting the ensemble size as 1 in the Chemprop API^4^ for the fair comparison. Hyperparameter tuning for the D-MPNN is listed in Table 3

Table 3 Hyperparameters optimized for D-MPNN

| Hyperparameters | Search space |
| --- | --- |
| Number of epochs | 30, 50, 100 |
| Batch size | 64, 128, 256 |
| Hidden size | 200, 300, 400 |
| Dropout | 0.1, 0.2, 0.3 |
| Depths | 2, 3, 4 |

# RDkit Features for Random Forest

The molecular feature used in random forest are generated from RDkit, with 208 features in total. All these features are listed below in order of Gini and permutation importance^5^.

Table 4 Molecular feature used in random forest, the rankings are based on the Gini importance and permutation importance.

| Rank | Feature name (sorted by Gini importance) | Feature name (sorted by permutation importance) |
| --- | --- | --- |
| 1 | VSA_EState8 | VSA_EState8 |
| 2 | TPSA | TPSA |
| 3 | SlogP_VSA5 | SMR_VSA5 |
| 4 | SMR_VSA5 | SlogP_VSA5 |
| 5 | NumHeteroatoms | NumHeteroatoms |
| 6 | NOCount | NOCount |
| 7 | EState_VSA7 | EState_VSA7 |
| 8 | MolLogP | MolLogP |
| 9 | PEOE_VSA6 | PEOE_VSA6 |
| 10 | EState_VSA10 | FractionCSP3 |
| 11 | Chi2n | Chi2n |
| 12 | FractionCSP3 | EState_VSA10 |
| 13 | fr_nitro | Kappa3 |
| 14 | Chi2v | Kappa2 |
| 15 | PEOE_VSA7 | Chi2v |
| 16 | VSA_EState3 | Chi0n |
| 17 | BCUT2D_MWLOW | Chi0v |
| 18 | Kappa3 | VSA_EState6 |
| 19 | VSA_EState6 | NumHAcceptors |
| 20 | BCUT2D_CHGLO | Chi1v |
| 21 | MaxPartialCharge | fr_nitro |
| 22 | Kappa2 | Chi1n |
| 23 | Chi1v | BCUT2D_MWLOW |
| 24 | Chi1n | VSA_EState3 |
| 25 | MinEStateIndex | EState_VSA6 |
| 26 | Chi0n | HallKierAlpha |
| 27 | NumHAcceptors | BCUT2D_CHGLO |
| 28 | Chi0v | PEOE_VSA7 |
| 29 | HallKierAlpha | Kappa1 |
| 30 | BCUT2D_MRLOW | MolMR |
| 31 | MolMR | MinEStateIndex |
| 32 | Chi3n | EState_VSA5 |
| 33 | EState_VSA6 | Chi3n |
| 34 | Kappa1 | BCUT2D_MRLOW |
| 35 | EState_VSA1 | VSA_EState2 |
| 36 | Chi3v | MaxPartialCharge |
| 37 | EState_VSA8 | EState_VSA1 |
| 38 | VSA_EState2 | RingCount |
| 39 | PEOE_VSA2 | Chi3v |
| 40 | fr_NH0 | EState_VSA8 |
| 41 | SlogP_VSA6 | PEOE_VSA8 |
| 42 | PEOE_VSA8 | BCUT2D_MWHI |
| 43 | Chi4v | BertzCT |
| 44 | BCUT2D_MWHI | SlogP_VSA6 |
| 45 | EState_VSA5 | PEOE_VSA2 |
| 46 | MinAbsPartialCharge | EState_VSA4 |
| 47 | Chi4n | Chi4v |
| 48 | VSA_EState4 | BalabanJ |
| 49 | BertzCT | Chi4n |
| 50 | BalabanJ | fr_NH0 |
| 51 | SlogP_VSA4 | MinAbsPartialCharge |
| 52 | EState_VSA4 | LabuteASA |
| 53 | FpDensityMorgan3 | SMR_VSA1 |
| 54 | LabuteASA | FpDensityMorgan3 |
| 55 | SMR_VSA1 | fr_bicyclic |
| 56 | SMR_VSA7 | VSA_EState4 |
| 57 | BCUT2D_CHGHI | SlogP_VSA4 |
| 58 | RingCount | VSA_EState9 |
| 59 | MaxEStateIndex | NumRotatableBonds |
| 60 | SMR_VSA10 | SMR_VSA7 |
| 61 | VSA_EState7 | SMR_VSA3 |
| 62 | BCUT2D_LOGPLOW | SMR_VSA10 |
| 63 | MinAbsEStateIndex | fr_Ar_N |
| 64 | MaxAbsEStateIndex | BCUT2D_LOGPHI |
| 65 | VSA_EState5 | Ipc |
| 66 | BCUT2D_LOGPHI | SlogP_VSA10 |
| 67 | FpDensityMorgan2 | FpDensityMorgan2 |
| 68 | BCUT2D_MRHI | BCUT2D_CHGHI |
| 69 | Ipc | HeavyAtomMolWt |
| 70 | EState_VSA2 | BCUT2D_LOGPLOW |
| 71 | NumRotatableBonds | FpDensityMorgan1 |
| 72 | MinPartialCharge | SMR_VSA6 |
| 73 | FpDensityMorgan1 | fr_nitro_arom |
| 74 | SlogP_VSA2 | PEOE_VSA13 |
| 75 | qed | EState_VSA2 |
| 76 | Chi1 | EState_VSA3 |
| 77 | HeavyAtomMolWt | VSA_EState7 |
| 78 | SMR_VSA3 | PEOE_VSA9 |
| 79 | fr_nitro_arom | Chi1 |
| 80 | MaxAbsPartialCharge | MinAbsEStateIndex |
| 81 | PEOE_VSA13 | MinPartialCharge |
| 82 | EState_VSA3 | NumValenceElectrons |
| 83 | MolWt | MaxEStateIndex |
| 84 | PEOE_VSA9 | Chi0 |
| 85 | ExactMolWt | MaxAbsEStateIndex |
| 86 | Chi0 | ExactMolWt |
| 87 | NumValenceElectrons | VSA_EState5 |
| 88 | fr_bicyclic | BCUT2D_MRHI |
| 89 | SMR_VSA6 | MolWt |
| 90 | SlogP_VSA10 | SlogP_VSA3 |
| 91 | VSA_EState9 | PEOE_VSA14 |
| 92 | VSA_EState1 | NumAromaticHeterocycles |
| 93 | SlogP_VSA3 | SlogP_VSA2 |
| 94 | PEOE_VSA14 | MaxAbsPartialCharge |
| 95 | fr_Ar_N | qed |
| 96 | PEOE_VSA1 | VSA_EState1 |
| 97 | PEOE_VSA10 | NumAromaticRings |
| 98 | SMR_VSA4 | SlogP_VSA8 |
| 99 | SlogP_VSA8 | PEOE_VSA10 |
| 100 | PEOE_VSA11 | NumAliphaticRings |
| 101 | EState_VSA9 | SMR_VSA4 |
| 102 | NumAromaticRings | fr_benzene |
| 103 | fr_benzene | NumAromaticCarbocycles |
| 104 | PEOE_VSA3 | PEOE_VSA1 |
| 105 | HeavyAtomCount | HeavyAtomCount |
| 106 | SlogP_VSA1 | EState_VSA9 |
| 107 | fr_N_O | PEOE_VSA3 |
| 108 | NumAromaticCarbocycles | PEOE_VSA11 |
| 109 | NumAromaticHeterocycles | NumAliphaticHeterocycles |
| 110 | NumAliphaticRings | SlogP_VSA1 |
| 111 | PEOE_VSA12 | fr_N_O |
| 112 | SMR_VSA9 | PEOE_VSA12 |
| 113 | fr_ether | fr_nitro_arom_nonortho |
| 114 | NumAliphaticHeterocycles | fr_ether |
| 115 | NHOHCount | SMR_VSA9 |
| 116 | fr_nitro_arom_nonortho | fr_C_O |
| 117 | NumHDonors | fr_C_O_noCOO |
| 118 | PEOE_VSA5 | NumSaturatedRings |
| 119 | fr_C_O_noCOO | NumHDonors |
| 120 | fr_C_O | fr_para_hydroxylation |
| 121 | PEOE_VSA4 | fr_NH1 |
| 122 | NumSaturatedRings | fr_hdrzine |
| 123 | fr_hdrzine | NumSaturatedHeterocycles |
| 124 | NumAliphaticCarbocycles | NHOHCount |
| 125 | fr_NH1 | SlogP_VSA11 |
| 126 | NumSaturatedHeterocycles | fr_methoxy |
| 127 | SlogP_VSA11 | PEOE_VSA4 |
| 128 | fr_ester | fr_oxime |
| 129 | NumRadicalElectrons | fr_aryl_methyl |
| 130 | fr_amide | NumAliphaticCarbocycles |
| 131 | NumSaturatedCarbocycles | fr_aniline |
| 132 | fr_aniline | fr_ester |
| 133 | fr_methoxy | fr_Nhpyrrole |
| 134 | fr_oxime | PEOE_VSA5 |
| 135 | fr_aryl_methyl | NumSaturatedCarbocycles |
| 136 | fr_para_hydroxylation | fr_Ar_NH |
| 137 | fr_allylic_oxid | fr_piperzine |
| 138 | fr_Al_OH | fr_amide |
| 139 | fr_ketone | fr_Ar_OH |
| 140 | fr_unbrch_alkane | fr_phenol_noOrthoHbond |
| 141 | fr_Ar_NH | fr_ketone |
| 142 | fr_Nhpyrrole | fr_allylic_oxid |
| 143 | fr_Al_OH_noTert | fr_Al_OH |
| 144 | fr_ketone_Topliss | fr_Al_OH_noTert |
| 145 | fr_NH2 | fr_phenol |
| 146 | SMR_VSA2 | NumRadicalElectrons |
| 147 | fr_nitrile | fr_ketone_Topliss |
| 148 | fr_pyridine | fr_NH2 |
| 149 | fr_Ar_OH | fr_unbrch_alkane |
| 150 | fr_phenol | fr_amidine |
| 151 | fr_phenol_noOrthoHbond | fr_nitrile |
| 152 | fr_piperdine | SMR_VSA2 |
| 153 | fr_ArN | fr_pyridine |
| 154 | fr_hdrzone | fr_Ar_COO |
| 155 | fr_Imine | fr_imide |
| 156 | fr_imide | fr_hdrzone |
| 157 | fr_piperzine | fr_COO |
| 158 | fr_amidine | fr_furan |
| 159 | fr_COO | fr_Ndealkylation1 |
| 160 | fr_Ndealkylation1 | fr_piperdine |
| 161 | fr_COO2 | fr_COO2 |
| 162 | fr_imidazole | fr_Imine |
| 163 | fr_urea | fr_Ndealkylation2 |
| 164 | fr_Ndealkylation2 | fr_tetrazole |
| 165 | fr_tetrazole | fr_ArN |
| 166 | fr_lactone | fr_aldehyde |
| 167 | fr_Ar_COO | fr_azo |
| 168 | fr_furan | fr_imidazole |
| 169 | fr_priamide | fr_lactone |
| 170 | fr_alkyl_carbamate | fr_morpholine |
| 171 | fr_azide | fr_alkyl_carbamate |
| 172 | fr_azo | fr_dihydropyridine |
| 173 | fr_Al_COO | fr_Al_COO |
| 174 | fr_nitroso | fr_urea |
| 175 | fr_morpholine | fr_quatN |
| 176 | fr_aldehyde | fr_azide |
| 177 | EState_VSA11 | fr_lactam |
| 178 | fr_quatN | fr_oxazole |
| 179 | fr_guanido | fr_nitroso |
| 180 | fr_term_acetylene | EState_VSA11 |
| 181 | fr_oxazole | fr_guanido |
| 182 | fr_epoxide | fr_term_acetylene |
| 183 | fr_dihydropyridine | fr_epoxide |
| 184 | fr_lactam | fr_sulfide |
| 185 | fr_barbitur | fr_sulfonamd |
| 186 | fr_HOCCN | fr_sulfone |
| 187 | fr_diazo | fr_isothiocyan |
| 188 | fr_isocyan | SlogP_VSA9 |
| 189 | VSA_EState10 | fr_prisulfonamd |
| 190 | fr_halogen | fr_thiazole |
| 191 | fr_thiophene | fr_thiocyan |
| 192 | fr_thiocyan | fr_thiophene |
| 193 | fr_thiazole | SlogP_VSA7 |
| 194 | fr_benzodiazepine | fr_phos_ester |
| 195 | fr_sulfone | SlogP_VSA12 |
| 196 | fr_sulfonamd | SMR_VSA8 |
| 197 | fr_sulfide | fr_isocyan |
| 198 | fr_SH | fr_phos_acid |
| 199 | SlogP_VSA9 | fr_C_S |
| 200 | SlogP_VSA7 | fr_HOCCN |
| 201 | SlogP_VSA12 | fr_SH |
| 202 | fr_phos_ester | fr_alkyl_halide |
| 203 | fr_phos_acid | VSA_EState10 |
| 204 | fr_isothiocyan | fr_benzodiazepine |
| 205 | fr_alkyl_halide | fr_diazo |
| 206 | SMR_VSA8 | fr_halogen |
| 207 | fr_C_S | fr_barbitur |
| 208 | fr_prisulfonamd | fr_priamide |

# Model comparison for small dataset performance

Although our FFiTrNet model uses a deeper discipline of molecular, having a better understanding of the molecule’s structure and property relation, making its prediction more accurate in both high-density and overall datasets, the main drawback is that the complexity of our model results in the requirement of more data to get this better result, because more parameters are needed to be trained in our model. If we simply use Huang & Massa dataset ^6^ (with only 109 materials) to train each model, our FFiNet and FFiTrNet models are hard to learn these patterns from the relatively small amount of data and could not show advantages over other simpler models. The performance of each model in the small dataset training is shown in Table 5. As expected, our model performs worse than other models which have simpler structures. Comparing to the Table 4 in the main acritical, our model’s performance greatly improves, showing the effectiveness of pretraining our model in the dataset to increase the accuracy.

Table 5 The test MAE, RMSE and *R^2^* for each model only use Huang & Massa dataset as training

| Models | MAE (g/cm^3^) | RMSE (g/cm^3^) | *R^2^* |
| --- | --- | --- | --- |
| RF | 0.0626 ± 0.0064 | 0.0848 ± 0.0197 | **0.5878 ± 0.1295** |
| GATv2 | 0.0656 ± 0.0056 | **0.0770 ± 0.0072** | 0.0177 ± 0.8424 |
| D-MPNNs | **0.0621 ± 0.0038** | 0.0877 ± 0.0091 | 0.3629 ± 0.5504 |
| FFiNet | 0.0839 ± 0.0040 | 0.0969 ± 0.0097 | -0.1733 ± 0.6029 |
| FFiTrNet | 0.0767 ± 0.0169 | 0.0893 ± 0.0203 | -0.0245 ± 0.4832 |

The best results are marked in **bold**, and the second-best results are underlined.

# Reference

1. Ren GP, Yin YJ, Wu KJ, He Y. Force field-inspired molecular representation learning for property prediction. *J Cheminform*. 2023;15(1). doi:10.1186/s13321-023-00691-2

2. Dosovitskiy A, Beyer L, Kolesnikov A, et al. An Image is Worth 16x16 Words: Transformers for Image Recognition at Scale. Published online October 22, 2020.

3. Pedregosa F, Varoquaux G, Gramfort A, et al. Scikit-learn: Machine Learning in Python. *Journal of Machine Learning Research*. 2011;12(85):2825-2830. http://jmlr.org/papers/v12/pedregosa11a.html

4. Yang K, Swanson K, Jin W, et al. Analyzing Learned Molecular Representations for Property Prediction. *J Chem Inf Model*. 2019;59(8):3370-3388. doi:10.1021/acs.jcim.9b00237

5. Breiman L. Random forests. *Mach Learn*. 2001;45(1):5-32. doi:10.1023/A:1010933404324/METRICS

6. Huang L, Massa L. Applications of energetic materials by a theoretical method (discover energetic materials by a theoretical method). *Int. J. Energ. Mater. Chem. Propul.* 2013, 12, 197–262.
